# Ectomycorrhizal Networks in the Anthropocene: From Natural Ecosystems to Urban Planning

**DOI:** 10.3389/fpls.2022.900231

**Published:** 2022-06-30

**Authors:** Louise Authier, Cyrille Violle, Franck Richard

**Affiliations:** ^1^CEFE, Univ Montpellier - CNRS - EPHE - IRD, Montpellier, France; ^2^Ilex Paysage + Urbanisme, Lyon, France

**Keywords:** plant-fungal interactions, ectomycorrhizal symbiosis, endophytic fungi, anthropogenic soils, forest soils, ecological succession, sylvigenetic cycle

## Abstract

Trees acquire hydric and mineral soil resources through root mutualistic associations. In most boreal, temperate and Mediterranean forests, these functions are realized by a chimeric structure called ectomycorrhizae. Ectomycorrhizal (ECM) fungi are highly diversified and vary widely in their specificity toward plant hosts. Reciprocally, association patterns of ECM plants range from highly specialist to generalist. As a consequence, ECM symbiosis creates interaction networks, which also mediate plant–plant nutrient interactions among different individuals and drive plant community dynamics. Our knowledge of ECM networks essentially relies on a corpus acquired in temperate ecosystems, whereas the below-ground facets of both anthropogenic ECM forests and inter-tropical forests remain poorly investigated. Here, we successively (1) review the current knowledge of ECM networks, (2) examine the content of early literature produced in ECM cultivated forests, (3) analyze the recent progress that has been made in understanding the place of ECM networks in urban soils, and (4) provide directions for future research based on the identification of knowledge gaps. From the examined corpus of knowledge, we reach three main conclusions. First, the emergence of metabarcoding tools has propelled a resurgence of interest in applying network theory to ECM symbiosis. These methods revealed an unexpected interconnection between mutualistic plants with arbuscular mycorrhizal (AM) herbaceous plants, embedding ECM mycelia through root-endophytic interactions. This affinity of ECM fungi to bind VA and ECM plants, raises questions on the nature of the associated functions. Second, despite the central place of ECM trees in cultivated forests, little attention has been paid to these man-made landscapes and in-depth research on this topic is lacking. Third, we report a lag in applying the ECM network theory to urban soils, despite management initiatives striving to interconnect motile organisms through ecological corridors, and the highly challenging task of interconnecting fixed organisms in urban greenspaces is discussed. In particular, we observe a pauperized nature of resident ECM inoculum and a spatial conflict between belowground human pipelines and ECM networks. Finally, we identify the main directions of future research to make the needed link between the current picture of plant functioning and the understanding of belowground ECM networks.

## Introduction

In most boreal, temperate and Mediterranean ecosystems, as in a part of tropical and sub-tropical forests, the canopies of few ectomycorrhizal (ECM) tree species dominate multi-layered communities of arbuscular mycorrhizal (AM) plants (Smith and Read, 2010; Tedersoo and Nara, 2010; Liang et al., 2020). ECM symbiosis shapes a wide variety of landscapes worldwide, from highly-preserved old-growth forests of the northern hemisphere (Smith et al., 2002; Spake et al., 2016), to emblematic oak savannas in Mediterranean biodiversity hotspots (Kennedy et al., 2012; Baptista et al., 2015), to highly artificialized tree plantations (Giachini et al., 2000) and orchards (Belfiori et al., 2012).

Belowground, ECM assist plant mineral nutrition by hydrolyzing natural polymeric compounds contained in litter and forest organic debris (Pritsch et al., 2011; Martin et al., 2016), and mobilizing soil water through absorptive hyphae (Lehto and Zwiazek, 2011). Based on their ability to densely colonize tree root systems, ECM mycelia establish hundreds of thousands of connections per square meter through short roots (Dahlberg et al., 1997; Taylor, 2002), from which thousands of kilometers of extrametrical mycelia are annually produced exploring soil for water, nutrients and new apices to colonize (Leake et al., 2004; Hagenbo et al., 2016), or provide physical support to plant–plant interactions in the soil.

Taxonomically, ECM mutualism is highly asymmetric, with a high diversity of some 20,000 species of Ascomycetes and Basidiomycetes linking only 6,000 species of Angiosperms and Gymnosperms within very few families (van der Heijden et al., 2015). In the ECM symbiosis, both the host and symbiotic species highly vary in their degree of specificity with their partners, from highly specialized (e.g., species in the Basidiomycete genera *Alnicola* and *Alpova* are strict associates of *Alnus* spp), to broad-host range species (e.g., the Ascomycete *Cenoccocum geophilum* associated with a wide range of Angiosperms and Gymnosperms; Bruns et al., 2002; Barham et al., 2014). These patterns underly the ability of ECM fungal diversity to interconnect ECM hosts through compatible dispersed inoculum across landscapes (Taudiere et al., 2015).

In multi-layered forests, the co-occurrence of plants from various mycorrhizal guilds constitutes the support for the establishment of common mycorrhizal networks (CMNs) linking canopies to the undergrowth through belowground mycelia. While AM networks interconnect roots from similar or different AM species of trees to shrubs and herbaceous plants (Wipf et al., 2019), fully autotrophs ECM trees exchange nutrients among each other (Klein et al., 2016) and with mixotrophic and mycoheterotrophic orchids (e.g., Li et al., 2021 for orchids) and ericaceous forest plants (Suetsugu et al., 2021).

Based on its ability to establish dense networks of hyphal connections among roots, ECM symbiosis strongly influences plant community composition and dynamics (Richard et al., 2009; van der Heijden et al., 2015; Nagati et al., 2019). Broad-host range species of fungi drive interspecific plant–plant interactions through shared mycelia (Beiler et al., 2010), which are then interconnected into CMNs. Reciprocally, hub species of ECM plants typically accumulate hyper-diverse communities of ECM fungi that co-occur on the local scale by their root systems (Tedersoo et al., 2012; Courty et al., 2016). In forest soils, these mycelial-mediated physical links among roots are involved in an underground carbon trade among co-occurring plant individuals (Simard et al., 2003; Klein et al., 2016). They constitute the below-ground facet of mycohetero- and mixo-trophic plant evolutionary lineages, whose species become established and survive along with ECM trees (Bidartondo, 2005; Selosse et al., 2006). From a phytocentric perspective, these interaction networks mediate positive soil feedbacks among co-occurring plants and drive ecological successions (Bever, 2002; Kennedy et al., 2012).

One of the best markers of the Anthropocene lies on the dramatic degradation of physical, chemical, and biological signatures of the pedosphere, and the rapid extension of human-made soil profiles in most parts of the world (e.g., *Anthrosols* and *Technosols*; Certini and Scalenghe, 2011). As organisms are highly dependent on soil physico-chemical conditions, ECM fungal communities are critical components of soil history by responding to short-term as well as long-term human impacts (Dupouey et al., 2002). Inevitably, the composition and dynamics of ECM community are profoundly influenced by forest management (Tomao et al., 2020) and anthropic disturbance derived from agronomic practices in man-made ecosystems (Olivera et al., 2014; Taschen et al., 2015), with cascading effects on the underlying interaction networks (Correia et al., 2021).

Since the early 2000s and the first evidence of the pivotal role of ECM CMNs in the dynamics of temperate plant communities during both primary (Nara, 2006a) and secondary successions (Simard et al., 1997), the ecological significance of ECM fungal-mediated interactions among plants has been a matter of ongoing debate (Bever et al., 2010; Birch et al., 2020). As a consequence, our understanding of the physical nature, functional boundaries, and trophic influence of ECM CMNs has considerably increased for the last decade, propelled by both the emergence of powerful metabarcoding tools and the deployment of a variety of experimental approaches in order to decipher underground ECM-based processes.

In this review, we provide an overview of major advances of our understanding of the ECM CMNs, focusing on the spatial extent, associated functions and effects of anthropic practices on mycelium sharing among plants. We evaluate the published literature and identify research gaps to determine promising research avenues along a gradient of anthropic footprint, extending from forests driven by spontaneous processes on one hand, to cultivated forests (see Glossary) and highly artificial urban ecosystems on the other. First, we provide a diachronic perspective on the role of ECM CMNs in natural ecosystems along ecological successions, with a focus on the sylvigenetic cycle (i.e., the ontogenetic cycle of the forest *sensu* Oldeman, 2012). Second, we mobilize the state-of-the-art concerning ECM network-based research in cultivated forests, including agroforests, to discuss the unexpected diversity of ecological guilds involved in ECM networks, and the consequences of our understanding of forest functioning. Third, we assemble current knowledge concerning ECM diversity patterns in urban soils, and discuss the challenge of conciliating ECM network-based services in cities and the development of human networks. Last, we propose a framework for future research across the gradient of ecosystems explored in the first three sections of the review.

## Ectomycorrhizal Networks Drive Plant Community Dynamics Along Ecological Succession

During ecological successions in temperate, Mediterranean, and boreal ecosystems, two dependent processes concomitantly unfold after disturbance. While aboveground communities of pioneer herbs and shrubs are progressively enriched by tree species, a microbiological switchover occurs belowground, from primarily arbuscular mycorrhizae associations (AM) on roots of early vegetation stages to their replacement by ectomycorrhizal association patterns on tree root systems (van der Heijden et al., 2015). In these ecosystems, the main tree families (i.e., Fagaceae, Betulaceae, Pinaceae, and Salicaceae; Taudiere et al., 2015) have the ability to associate with ECM fungi. Contrastingly, only two families of shrubs (i.e., Cistaceae and Ericaceae *pro parte*; Comandini et al., 2006; Richard et al., 2009) are ectomycorrhizal. In this section, we successively discuss (1) the networking role of these ecologically pivotal shrub species that endure the transition between VA and ECM plant communities and (2) our knowledge of the role of ECM CMNs during the sylvigenetic cycle, from seedling establishment to the senescence of forest trees.

### ECM Networks Mediate Plant–Plant Interactions in Early Successional Stages

Our knowledge of the ecological significance of ECM networks along succession almost entirely relies on data accumulated in boreal and temperate ecosystems of the Nearctic and palearctic regions (but see Ramanankierana et al., 2007 for a tropical perspective). In this documented area, and using a combination of *in situ* measurements, soil bioassays, and seedling transplantation, a consistent pattern of CMN-mediated nurse effect by shrubs on ECM late successional tree species has been reported in northern America (*Arbutus menziesii* vs. *Pseudotsuga menziesii*; Molina et al., 1997; Horton et al., 1999; Kennedy et al., 2012); *Helianthemum bicknellii* vs. *Quercus spp*; (Dickie et al., 2004), southern Europe (*Arbutus unedo* vs. *Quercus ilex*; Richard et al., 2009) and eastern Asia (*Salix reinii* vs. *Betula ermanii* and *Larix kaempferi*; Nara and Hogetsu, 2004; Nara, 2006b).

In the different investigated case studies, the highlighted CMNs-based facilitation process similarly lies on two complementary characteristics regarding the ecology of the nurse shrub species. First, ECM nurses have the ability to hold a prominent place in early stages of ecological succession, by either surviving after disturbance (Nara and Hogetsu, 2004) or establishing as a pioneer ECM species in a plant matrix exclusively composed of VA species (Dickie et al., 2004; Richard et al., 2009; Kennedy et al., 2012). Second, beyond this role of ecological hinge, ECM nurses act as inoculum relays for late successional tree species by accumulating diversified communities of ECM fungal partners on their roots, including generalist species which increase the potential for connection into common mycorrhizal networks. In previous research, the efficiency of CMNs in early vegetation stages has been evaluated by measuring the ability of these hub species to sustain the mycorrhization of the benefactor species (e.g., Kennedy et al., 2012) and by evaluating the subsequent gain on their growth, nutrient uptake and survival (e.g., Nara and Hogetsu, 2004).

**Figure 1:**
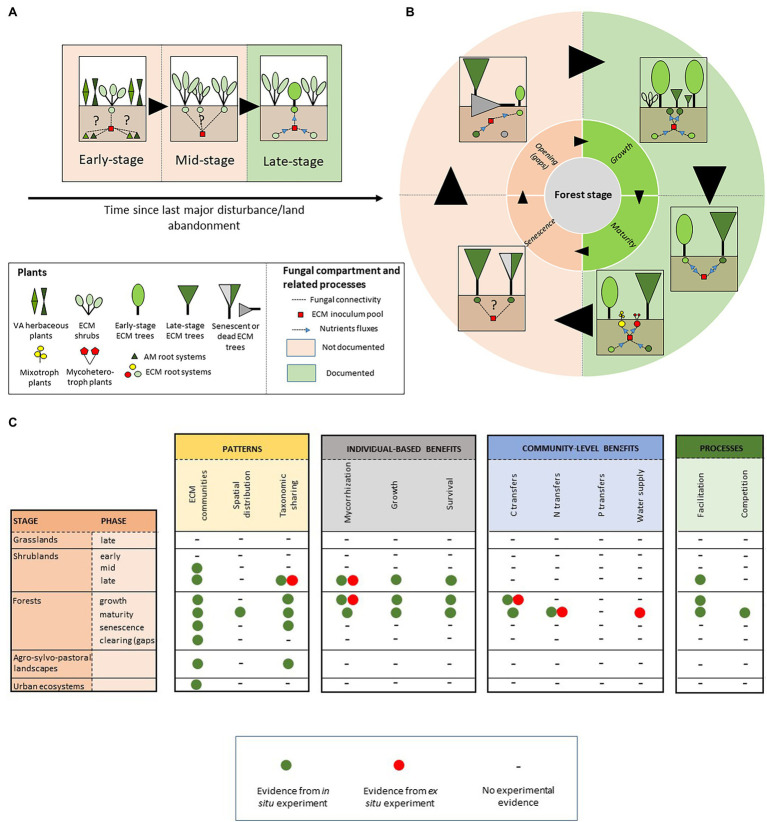
Schematic representation of ectomycorrhizal-based networks **(A)** across early ecological succession and **(B)** during sylvigenetic cycle. Dashed lines indicate plant–plant interactions through ECM CMNs. Blue arrows indicate nutrient transfers through ECM CMNs. **(C)** Summary of ECM network-related effects documented along the chronosequence.

Understanding the drivers of tree seedling establishment and survival is a central question in forest ecology, and a major challenge for forest and land managers. As a consequence, most research has been focused on the understanding of the role of ECM CMNs at the end of a shrub-dominated stage, when ECM tree species spontaneously establish in matrices of ECM nurse shrubs. These works overlook the major part of the chronological sequence since disturbance (Figure 1A). In particular, we still know little about the early ecological bases of early ECM networks, i.e., upon ECM shrub establishment in VA plant communities (Figure 1A). Interconnections among roots of VA and ECM plants through ECM-based mycelia may occur, as suggested by recent studies showing a possible dual [ECM + endophytic; see Glossary] niche for species of various lineages of ECM fungi in both asco- (e.g., *Tuber melanosporum* and *Tuber aestivum*; Schneider-Maunoury et al., 2018, 2020) and basidiomycetes (e.g., *Rhizopogon* spp.; Toju et al., 2018). The functional role of such plant–plant interactions is not clearly understood, and may include nutrient transfer from VA to ECM plants (see Taschen et al., 2020 for *Tuber melanosporum*), with possible consequences on the establishment and survival of ECM shrubs in stressful environments. Identifying both ECM fungal and VA plant hubs involved in these tripartite interaction networks may provide insights into the biotic dimension of both ECM plant and fungal fundamental niches, and offer promising perspective on restoration ecology, similarly to nurse-based processes of restoration developed on post-mining degraded ecosystems (Demenois et al., 2017).

Furthermore, the belowground facet of the functioning of highly resilient shrub populations is still poorly known in the later succession stages (Figure 1A). In particular, the role of mycelium sharing among conspecific ECM shrubs, as a possible mechanism underlying the drought tolerance and adaptation of these communities to disturbance remains poorly addressed. This is the case for Cistaceae, an emblematic family of evergreen shrubs dominating Mediterranean-type landscapes at low elevation (Guzmán and Vargas, 2009). Based on fruitbody surveys, there is evidence to suggest that Cistaceae host highly diversified and species-rich ECM fungal communities (Comandini et al., 2006; Loizides, 2016; Leonardi et al., 2020), yet little is known of their belowground patterns and functioning. Exploring the topology (see Glossary) of the corresponding CMNs, and their response to drought and disturbance in widely distributed monospecific stands, may contribute to a systemic understanding of the belowground adaptation processes of Mediterranean plants.

### ECM Networks Sustain Nutrient Exchange Among Trees Along the Sylvigenetic Cycle

As forests age, two highly dependent community dynamics concomitantly occur. Thus, the progressive replacement of short-lived early-stage ECM trees (e.g., Betulaceae, Salicaceae, and Pinaceae pro parte) by long-lived shade-tolerant dryads aboveground (e.g., Fagaceae and Pinaceae *pro parte*) is accompanied by a marked shift in the composition of associated belowground communities (Bruns et al., 2002; Taudiere et al., 2015). On tree roots, while early-stage trees host poor ECM fungal communities, dryads associate with multiple fungal species, and progressively accumulate species on their roots (e.g., Smith et al., 2002; Richard et al., 2005), with specialization degree of fungal partners increasing with tree aging (Bruns et al., 2002; Barham et al., 2014; Taudiere et al., 2015). Meanwhile, the underground composition of early-stage ECM fungal communities reciprocally drives the recruitment of late-stage plants. On roots of pioneer trees, broad host range fungal hubs transfer nutrients to late-stage trees through CMNs (Teste and Simard, 2008; Nagati et al., 2019), increasing their establishment success through soil feedbacks (Liang et al., 2020), most notably in arid environments (Bingham and Simard, 2012; Montesinos-Navarro et al., 2019).

Simard’s pioneer work (Simard et al., 1997) propelled intensive research on the understanding of the role of ECM CMNs along forest sylvigenetic cycle. In particular, the ability of CMNs to mediate the recruitment of late-stage tree seedlings under the canopies of pioneer species has been largely explored. Interplant nutrient transferring through ECM CMNs is now considered as one of the most fascinating mechanisms involved in shade tolerance of late-stage tree species, which establish during forest ontogeny (Figure 1B; Teste and Simard, 2008; Simard et al., 2012), and partially and fully heterotrophic plant lineages living in the undergrowth (Figure 1B; Li et al., 2021). However, we still know little about the ecological significance of these trophic interactions among neighboring trees at the forest ecosystem level. Consistent evidence suggests that plant–plant interactions through shared ECM mycelia may play a central role in the performance of tree seedlings in mature forests in boreal (Nagati et al., 2019), temperate (Teste and Simard, 2008), Mediterranean (Egerton-Warburton et al., 2007), and tropical climates (Liang et al., 2020). The consequences of these interactions on the long-term ecosystem dynamics still need to be tested. For instance, community patterns and dynamics suggest that CMNs may drive soil-mediated positive feedback loops (Bever et al., 2010), which in turn may shape directional succession induced by climate change in boreal ecosystems (Deslippe and Simard, 2011; Nagati et al., 2019). In natural forests, the role of CMNs-based, long-term process in forest dynamics still need to be evaluated. For instance, in Mediterranean old-growth forests, shade-tolerant *Q. ilex* seedlings survive in light-restricted conditions for decades, until the senescent phase and the advent of canopy openings (Panaiotis et al., 1997). In gaps, these pre-established individuals avail of these conditions to emerge from the shrubby matrix and renew tree populations (Panaïotis et al., 1998). In the soil of senescent *Q. ilex* forest patches, seedlings and old *Q. ilex* individuals share high diversity of ECM symbionts on their roots (Richard et al., 2005). Along this well-described sylvigenetic cycle, quantifying nutrient transfers among conspecific individuals during mature and senescent phases, may advance our knowledge of the efficiency of CMNs-based interaction in forest regeneration.

The mechanisms underlying CMNs effect on forest dynamics are not fully understood. In particular, our knowledge of the nature and the ecological importance of transferred resource among co-occurring hosts remains partial. However, CMNs undoubtedly drive carbon transfer along reversible source-sink avenues at the local scale (Teste et al., 2009; Song et al., 2015; Cahanovitc et al., 2022). One of the most spectacular demonstration of this mechanism consists of carbon transfers among adult trees in temperate mixed conifer-hardwood forests (Klein et al., 2016). This finding is the first to reveal bidirectional nutrient exchanges among co-occurring ECM mature trees, with a significant amount of the carbon accumulated on roots of adult trees being transferred from neighboring donors. These transfers may be mainly based on fungal genera which typically dominate in mature forest stands (i.e., *Russula*, *Cortinarius*, *Lactarius*, and *Tricholoma*; Courty et al., 2005; Richard et al., 2005). Contrastingly, the influence of ECM CMNs on nitrogen and phosphorus sharing among co-occurring individuals remains less obvious (Figure 1C). However, ECM network-mediated nitrogen fluxes have been evidenced between co-occurring ECM tree species with contrasted nitrogen-acquisition strategies (He et al., 2006; Teste et al., 2015). The importance of these nutrient transfers on forest functioning is still under debate. Nevertheless, recent data suggest that they may drive feedback loops which promote tree population dynamics in monospecific tropical forests (Corrales et al., 2016). More generally, nutrient transfers through ECM CMNs are highly suspected to shape soil feedbacks and drive either cyclic or directional succession in the corresponding plant communities, from the tropics to arctic ecosystems (Deslippe and Simard, 2011; Kadowaki et al., 2018; Montesinos-Navarro et al., 2019; Liang et al., 2020). The knowledge accumulated during the last decade revealed an unexpectedly large biogeographical range and functional significance of ECM network-based ecosystem processes along forest ontogeny. However, research gaps still persist on both descriptive and functional facets of ECM networks in forest ecosystems. In particular, the role of ECM CMNs at the end of the sylvigenetic cycle, and particularly in natural canopy gaps, still need to explored (Figure 1C).

The belowground architecture of root systems of ECM trees, and the spatial distribution of the associated ECM fungal symbionts are poorly predicted by the vertical projection of the corresponding canopies (Lian et al., 2006; Taschen et al., 2015). Likewise, documenting the spatial patterns of plant–plant physical links through CMNs provide apparently counter-intuitive connections among highly spatially-distant individuals and determine unexpected hub individuals among populations (Teste et al., 2009), with consequences on forest management when tree individuals sustain production of associated resources (e.g., Lian et al., 2006 for Matsutake forests). At the local scale, we still know little about the distribution of links between plant and fungal individuals. However, first evidence from temperate *P. menziesii* uneven forests strikingly revealed the ability of ECM CMN to interconnect most co-occurring plant individuals with each-other (Beiler et al., 2010). In those ecosystems, and based on the use of multi-locus, microsatellite DNA markers, Beiler et al. (2015) revealed the nested topology of *Rhizopogon* ssp.-based ECM networks, suggesting a potential role in the resilience of tree population through the prevention of cascading effect following tree death. To the best of our knowledge, most ECM ecosystems still await for similar systemic (i.e., network-based) assessments of their belowground functioning, despite the promising potential of networks-derived metrics for forest management and conservation (Taudiere et al., 2015).

## In Cultivated Ecosystems, Anthropic Practices Affect Ectomycorrhizal Networks

Only 2% of vascular plant species are ectomycorrhizal (van der Heijden et al., 2015; Brundrett and Tedersoo, 2020). The majority of timber, softwood lumber and construction wood traded worldwide derives from this low percentage of vascular plants (Smith and Read, 2010). Most emblematic agro-sylvo-pastoral landscapes in the northern hemisphere are shaped by these plant species (e.g., Conedera et al., 2016 for an illustration with the domesticated ECM tree *Castanea sativa*). As a consequence, the effect of sylvicultural practices on ECM fungal communities, and the nature of adapted forest management practices to maintain diverse ECM communities has been intensively debated (see Tomao et al., 2020 for a review). Here, we successively discuss the current knowledge of the effect of human practices on ECM fungal networks in two main contexts. We first consider cultivated ecosystems as a wide variety of ECM forest types cultivated for timber production (see Glossary). Second, we review the state of the art in agroforests, i.e., in ECM socio-ecosystems where timber production is associated with a wide variety of human services, including crop and fruit productions (e.g., Dehesas, Montados, and planted orchards; Grove and Rackham, 2001).

### Anthropic Disturbances Shape ECM Communities and Networks in Cultivated Forests

In cultivated ECM forests, sylvicultural practices generally consist of interrupting the sylvigenetic cycle at the maturity phase, truncating the end of both aboveground and belowground ecological successions. In most documented ecosystems dominated by either angiosperms or gymnosperms, both selective tree logging and clearcutting have been reported to induce marked compositional change in ECM fungal communities. The higher the intensity of practices, the higher are the deleterious effects on ECM diversity (Sterkenburg et al., 2019). Specifically, it has been shown that ECM community diversity positively responds to tree diversity, basal area and canopy cover in cultivated forest systems (Tomao et al., 2020; but see Craig et al., 2016; Spake et al., 2016 for case study-dependant contrasted responses). As a practical consequence, the retention of forest patches (Kranabetter et al., 2013; Varenius, 2017), green trees (Sterkenburg et al., 2019), and to a lesser extent coarse woody debris (Walker et al., 2012) at harvest time have been reported as efficient compensatory measures to maintain ECM fungal diversity in cultivated ecosystems. If direct effect of tree removal are widely documented, indirect effect deleterious of forest logging have been also reported, including environmental change (Varenius, 2017), soil compaction (Hartmann et al., 2014), and soil amendment (Olivera et al., 2014; Almeida et al., 2018).

The effect of forest management practices on ECM CMNs have been poorly investigated so far. However, the accumulated knowledge during the last decade in forest ecosystems gives a theoretical framework to analyze the response of plant-fungi bipartite networks to forest practices. From a plant-centered perspective, the reported pauperization effect of forest exploitation on ECM communities may alter the topology of ECM networks. Early evidence of age influence on network topology already exists: using high-throughput sequencing of soils, Correia et al. (2021) reported contrasted ECM network topologies along a chronosequence of *Fagus sylvatica* forest establishment, plant nodes in long-established forests presenting higher numbers of connection links than in recent patches. From a fungal perspective, silvicultural practices tend to reduce the number and diversity of available plant nodes for fungal genets. As a consequence, tree logging may decrease the linkage density of ECM CMNs in cultivated stands. When considering the highly saturated and nested nature of ECM CMNs (Beiler et al., 2010; Taudiere et al., 2015), one may suggest consequences of tree logging on ECM network-mediated processes in forest ecosystems, including affected tree regeneration dynamics (Pec et al., 2020). On the basis of scarce preliminary works, further research is needed to illuminate the relationships between the complexity and the stability of ECM CMNs. Cultivated forests are also ideal candidate systems to study the links between the stability of ECM CMNs and the resilience of forest ecosystems.

### ECM Fungi Shape Complex Interaction Networks in Tree Savannas

Ectomycorrhizal trees dominate millions of hectares of anthropogenic landscapes in temperate and Mediterranean ecoregions, where trees, pastures, and croplands amalgamate in complex and species-rich mosaics called tree savannas (Grove and Rackham, 2001; Moreno and Pulido, 2009; see Glossary). Most ECM tree species contribute to the current highly diversified physiognomy of these systems, but the most emblematic ones are dominated by various species of oaks (e.g., from *Quercus velutina* dominating north America oak openings and typical *Quercus suber* dehesas, which cover one-eighth of Spain), sweet chestnut (e.g., *C. sativa* montados currently covering one-sixth of the area of Portugal), or larches and pines, which dominate multifunctional transitional landscapes between subalpine forests and alpine shrublands across Europe.

Because tree savannas play important ecological, social, and economic roles for societies (e.g., Conedera et al., 2016), understanding their functioning and analyzing their biodiversity patterns has been subject of much attention (Bugalho et al., 2011). Within their soils, these ecosystems concentrate some of the richest ECM communities described so far (Tedersoo et al., 2006; Morris et al., 2008; Baptista et al., 2015; Reis et al., 2018) and harbor highly specific fungal assemblies dominated by ascomycetes, in response to a unique combination of environmental and anthropic drivers (Dickie et al., 2009). Despite the primary importance of tree savannas for conservation, very little is known about their belowground functioning, and ECM-mediated plant–plant interactions remain poorly explored in most of them.

**Figure 2:**
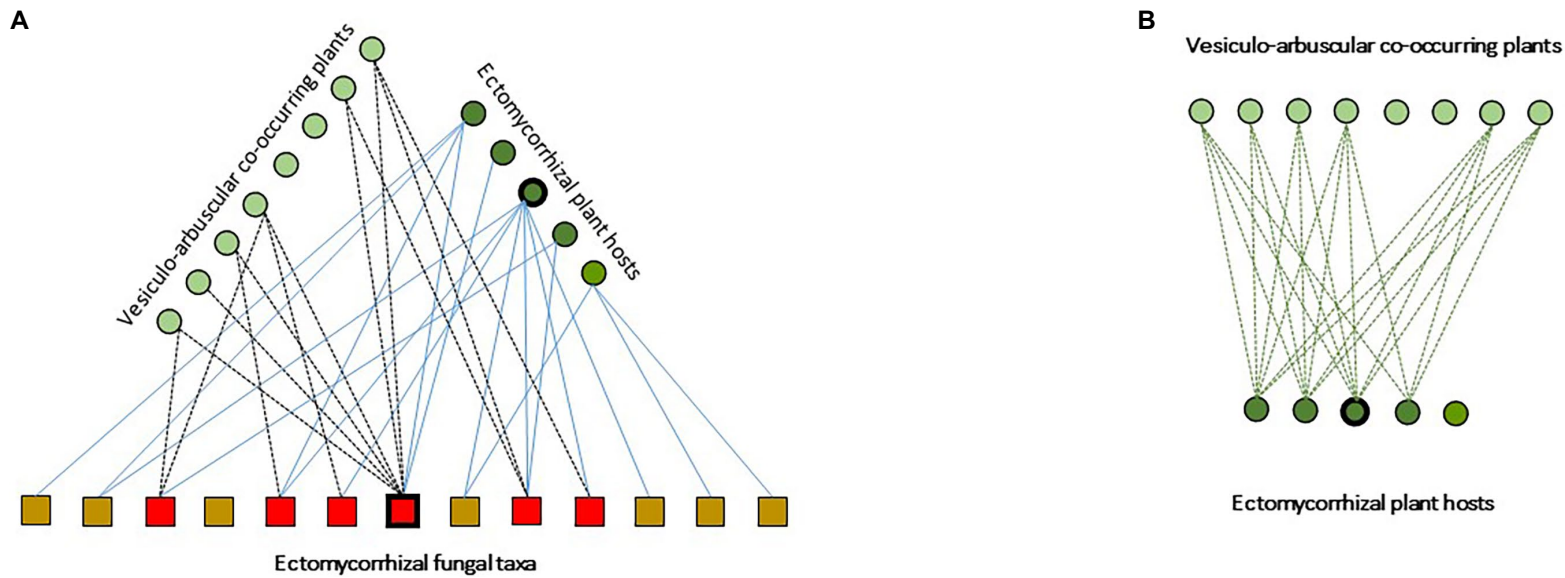
Schematic representation of ectomycorrhizal-based multipartite networks in *Tuber* s*sp.* temperate agroforests, from Schneider-Maunoury et al. (2018) modified. **(A)** Species of co-occurring ectomycorrhizal and arbuscular mycorrhizal plants are represented by dark and clear green dots, respectively. The different taxa of ECM fungi are represented by squares. Interaction hubs are indicated by thickened outlines (here, *Quercus ilex* and *Tuber melanosposrum*). Solid lines indicate plant-fungi ECM interaction links. Dotted lines indicate interaction links between ECM fungal tava and VA plants. **(B)** Schematic representation of the corresponding projected VA-ECM plant network.

Within this panorama, spontaneous oak savannas and planted orchards for the production of the emblematic black truffle (*Tuber melanosporum*) are an exception. Indeed, during the last decade, very few ECM communities received as much attention as *T. melanosporum* truffle grounds. In these transient ecosystems, a set of practices by truffle growers shape species-rich ECM communities, typically dominated by lineages of ascomycetes (e.g., Pyrenomataceae, Tuberaceae, and Helvellaceae) and early-stage lineages of basidiomycetes (e.g., Thelephoraceae) in both spontaneous and planted truffle grounds (e.g., Belfiori et al., 2012; Taschen et al., 2015). In these communities, *T. melanosporum* and few species in the early-stage lineages of basidio- (i.e., Thelephoraceae, Sebacinaceae, *Inocybe* spp., and *Scleroderma* spp.) and ascomycetes (i.e., *Cenoccocum geophilum* and Pyronemataceae), simultaneously associate as mutualists with their host through ECM root tips, and interact as fungal endophytes on the roots of most VA plant species established under host canopies (Schneider-Maunoury et al., 2018, 2020; Figure 2A). In these systems, the topology of the below-ground fungal interaction networks remains undescribed (Figure 2A). Similarly, the ecological consequences of the “beyond forest edge” spatial extent of ECM CMNs remains poorly addressed (Taschen et al., 2020). In particular, the ability of ECM fungi to transcend their host range and to establish as endophytes in the tissues of AM plants (Taschen et al., 2020) still need to be examined under a functional perspective. These remarkable findings pave the way for pursuing the exploration of ECM-VA plant interaction mediated by shared ECM fungal hubs (Figure 2B), to provide systemic views of forest functioning.

## ECM Common Mycorrhizal Networks in Urban Soils: Headache in a Saturated Below-Ground

In anthropogenic urban ecosystems, a few ECM trees and shrubs (e.g., *Tilia*, *Pinus*, *Quercus*, and *Cistus*) hold a prominent place among a wide range of plant species adapted to the environmental conditions and physical constraints of cities (Bainard et al., 2011; Williams et al., 2015; Jenerette et al., 2016). In urban green spaces, the selection of plant species by urban land managers is in priority driven by socioeconomic constrains (Hope et al., 2003). In particular, a panel of aesthetical traits, including blooming intensity, flower size and color, shape patterns of urban tree diversity in cities (Kendal et al., 2012; Conway and Vander Vecht, 2015; Jenerette et al., 2016; Goodness, 2018). To a lesser extent, ecophysiological traits of species also matter, in particular plant tolerance to drought or freeze (Jenerette et al., 2016; McPherson et al., 2018). On the other hand, plant-related extended phenotype functions (Dawkins, 2016), such as fungal-mediated adaptation to drought through mycorrhizal symbionts, or nutrient uptake based on connection with neighboring individuals through ECM CMNs, still struggle to be included in selection processes among plant candidates.

### Urban Soils Host Pauperized ECM Fungal Communities

The composition of soil fungal communities in urban soils remains largely unexplored (Delgado-Baquerizo et al., 2021). Published research consistently shows eroded patterns of ECM diversity in cities, as compared to forest ecosystems (Bainard et al., 2011; Karpati et al., 2011; Martinová et al., 2016). In particular, a low ECM species richness has been observed at the local scale, on roots of planted trees in city parks (Bainard et al., 2011; Karpati et al., 2011), streetscapes (Bainard et al., 2011), and private residential properties (Bainard et al., 2011), with an unusual dominance of hypogeous fungi (e.g., species in the Tuberaceae) and an underrepresentation of ECM families which typically dominate in forest soils (i.e., *Russulaceae*, *Inocybaceae*, and *Cortinariaceae*; Martinová et al., 2016; Hui et al., 2017; Van Geel et al., 2018). Interestingly, these pauperized communities show a high ability to colonize root systems, with similar ECM colonization rates on roots in urban soils as comparted to forests (Timonen and Kauppinen, 2008; Bainard et al., 2011; Tonn and Ibáñez, 2017). This pauperized nature of ECM communities in urban soils and subsequent homogenization of ECM fungal communities among cities on a continental scale (Schmidt et al., 2017; Delgado-Baquerizo et al., 2021), question about the mechanisms driving the observed compositional change and the consequences of species shift on urban ecosystem functioning. Three main mechanisms may concomitantly drive the observed patterns.

**Figure 3:**
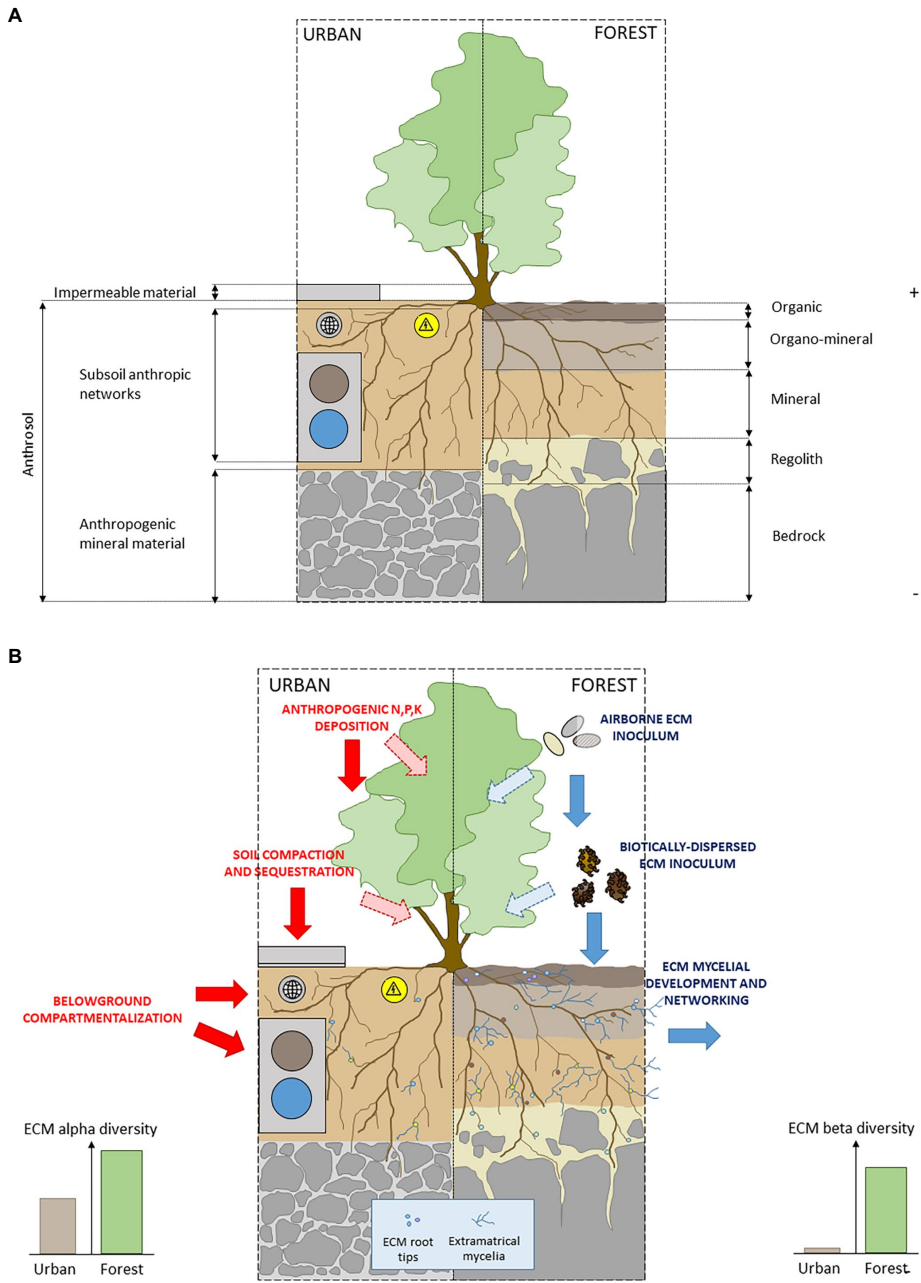
**(A)** Schematic representation of pedogenic conditions for the development of ectomycorrhizal plants in urban and forest soil conditions and **(B)** schematic representation of ECM network-based mechanisms and anthropic filters in urban and forest soils conditions.

First, a deleterious effect of urban environment on both the dispersion (by physical barriers among poorly connected and highly fragmented habitats) and establishment (by unsuitable physico-chemical conditions in anthrosols; Figure 3A) are likely to act as filters on the potential communities of airborne and biotically dispersed ECM inoculum (Kasprzyk and Worek, 2006; Koide et al., 2011). In particular, sealing of urban soil surface may impact ECM dispersion from adjacent forests to city streets, with negative effects on ECM richness and diversity in urban soils (Martinová et al., 2016; Figure 3A).

Moreover, the low alpha diversity of ECM communities in cities may reflect the specific physicochemical properties of urban soils (Newbound et al., 2012; Lüttge and Buckeridge, 2020). Thus, the role of soil chemical signature as one of the main drivers of the composition, diversity, temporal dynamics and spatial patterns of ECM fungal communities has been widely documented in forest ecosystems (e.g., Lilleskov et al., 2011 for a review; Courty et al., 2016 for a functional perspective). Unsurprinsingly, in urban as in forest soils, the vegetative development (Olchowik et al., 2021), and the species richness (Newbound et al., 2012; Martinová et al., 2016; Van Geel et al., 2018) of ECM communities are negatively affected by soil alkalinity, and positively respond to organic matter and moisture content (Van Geel et al., 2018). The consistent pattern of ECM richness decrease and the composition drift observed in urban soils have then to be considered in light of (1) currently widespread practices along city roads, including deicing salts contributing to the alkalization of urban soils (Czerniawska-Kusza et al., 2004) and (2) the critically low organic matter content in city soils, in particular in sealed contexts (Scharenbroch et al., 2005; Alzetta et al., 2012), and its deleterious consequences on the establishment of species with affinities for organic soils (e.g., Genney et al., 2006; Kranabetter et al., 2009).

Last, the simplification of ECM communities in urbanized contexts may be a consequence of anthrosol assembling process in urban green spaces. This highly artificial growth matrix generally consists of a unique soil horizon intercalated between a deeper layer of mineral anthropogenic substrate, and a cover of impermeable materials (Rodríguez-Espinosa et al., 2021; Figure 3A). The specific physical soil properties of anthrosols (Lorenz and Lal, 2009) combined with surface sealing, drastically affects biological processes (Lu and Weng, 2006; Martinová et al., 2016; Salvati et al., 2016; Hui et al., 2017) limiting the differentiation of soil profile into distinct horizons during pedogenesis (Hui et al., 2017; Figures 3A,B). As a consequence, anthrosols remain unsuitable habitats for the vertical stratification of ECM diversity from organic surface to the underlying mineral horizons (Rosling et al., 2003; Genney et al., 2006), by penalizing hyper diverse ECM sub-communities with affinities for surface organic layers (Richard et al., 2011).

### Traffic Jam in Urban Soils

During the last decade, restoring ecological networks in urban areas (Ignatieva et al., 2011) has become a priority for urban landscape planners, in order to provide suitable habitats for organisms and re-establish connectivity among fragmented meta-populations (Peng et al., 2017). The development of urban green corridors connecting habitat patches has been successfully developed in cities worldwide, as an efficient strategy to maintain high levels of alpha diversity (see Glossary), in particular for motile organisms (Beninde et al., 2015).

Contrastingly, fixed organisms such as plants and their fungal root associates still await to benefit from an adequate declination of network-based strategy for the conservation of dedicated belowground mutualistic interactions and their underlying functions. In particular, the topology of pauperized ECM CMNs in urban soils remains poorly described, the network-based signature of plant adaptation to urban environment still need to be explored, and the role of ECM CMNs for plant species coexistence in cities still need to be understood.

Yet, in urban areas as in natural ecosystems, there is strong evidence for a pivotal role of ECM colonization and diversity for promoting nutrient uptake (Newbound et al., 2010), acclimatation to hydric stress (Fini et al., 2011), tolerance to salt exposition (Zwiazek et al., 2019), and survival of ECM plants (Tonn and Ibáñez, 2017). Despite this pivotal role of ECM symbiosis, planning practices fail at integrating plant–plant ECM interconnexion through CMNs in urban design. At the individual level first, urbanization imposes to belowground counterparts of trees undersized dedicated soil volumes in compartmentalized planting pits (Day et al., 2010; Figure 3A). At the plant population/community level, the organization of cities constrains root development patterns along green linearities bordering transport networks (Levinson, 2012), anthropic pipelines (e.g., electrical, water, internet; Galle et al., 2019), and carriageway stabilizing materials (Randrup et al., 2001). Moreover, from a temporal perspective, high turnover of land-use of volatile urban landscapes hampers belowground mycelial dynamics by inducing severe soil disturbance regime (Moore, 2008; Lüttge and Buckeridge, 2020).

All in all, and further considering the temptation to consider urban underground space as an opportunity to compact city development (Cui et al., 2021), there is clear evidence that the establishment of functional ECM CMNs in cities may collide with the current geographical conceptualization of urban underground in the cities of the Anthropocene (Qihu, 2016; Connor and McNeill, 2022).

## Perspectives and Conclusions

The understanding of ectomycorrhizal interaction networks is a growing body of research. Their deterioration by human impacts is an overlooked marker of the anthropogenic footprint on terrestrial ecosystems. The pedosphere is particularly impacted by the rise to dominance of anthropic disturbances (Certini and Scalenghe, 2011). Here, we aimed at providing a comprehensive overview of the recent advances on our understanding of ECM CMNs in a wide range of ecosystems differing in the intensity of anthropic influence, in order to draw up a framework of timely research avenues. From this analysis, three issues should be primarily addressed.

### Knowledge Gaps in Natural Gaps

During the last decade, the development of metabarcoding tools propelled an unprecedented level of knowledge of belowground fungal patterns in forest soils, and enlarge perspectives on fungal assembly at both local (e.g., Baptista et al., 2015) and global (Tedersoo et al., 2014, 2020) scales. Despite significant progress been made, several gray areas persist. First, the topology of CMNs at the establishment time of pivotal ECM shrubs (Figure 1A) and the affinity of ECM fungi toward AM plant hosts, remain fascinating and poorly documented issues in ecology. Second, studies investigating network-based dynamics in soils of natural canopy gaps are surprisingly lacking. Yet, understanding the remobilization of late-stage trees inoculum heritage in soil by pioneer species to initiate a new sylvigenetic cycle (Figure 1B) is a central question for forest ecologists and managers. Last, there is urgent need to assess the effect of forest management practices on the topology of ECM networks since forest ecosystems face dramatic change through the combined effects of local impactful disturbance and global change.

### Taxonomic Limits and Spatial Patterns of ECM CMNs

One major recent advance in our knowledge of ECM symbiosis is the ability of various ECM fungal lineages to interconnect between their hosts and VA plants, in both forests (Toju et al., 2018) and tree savannas (Schneider-Maunoury et al., 2020). This unexpected porosity among ecological guilds of plants raises the questions of (1) the spatial limits of ECM CMNs through physical links among involved plant individuals and (2) the taxonomic basis of these interactions in multi-layered plant communities. Moreover, the role of these mixed (ectomycorrhizal-endophytic) connections by polyvalent fungal species for forest community dynamics necessitates more effort to be fully understood (Taschen et al., 2020). This finding suggests to re-examine, from a biotic and below-ground perspective, the mechanisms involved in the particularly widespread nursing effect of VA plants for ECM trees.

### Toward Soil Corridors for Underground Cities

Our synthesis reveals that patterns and functions of ECM CMNs in urban soils are poorly understood. However, given the rate of soil artificialization in cities worldwide (Scalenghe and Ajmone-Marsan, 2009; Just et al., 2018), there is crucial need to consider urban soils and their living organisms as a valuable resource for citizens in the Anthropocene. From a landscape management perspective, cities may be ideal candidates for the establishment of ECM network-based plant synergies in artificial green spaces, by designing interconnected planted pits along soil continuums, to maintain functional and multi-scaled ECM CMNs within *beige* corridors.

In such corridors, mimicking ECM networks established in natural ecosystems may be a promising avenue for urban planners to design functionally efficient artificial green spaces. First, in the cities of the Anthropocene, the composition of plant communities may (1) favor species with high number of associated ECM fungal species (i.e., interaction hub species such as Fagaceae; Taudiere et al., 2015) to increase the alpha diversity of ECM fungal inoculum, (2) associate nurse plants and late-stage benefactor species in multi-layered designs (Figure 1), and (3) promote synergies between AM and ECM plant guilds through endophytic interaction (see Glossary; Figure 2). In these artificial ecosystems, tree populations may be uneven-aged, to increase the complexity of ECM networks by promoting the emergence of interaction-hub tree individuals (i.e., aged individuals with high number of linked fungal individuals; Beiler et al., 2010), and subsequently favor the ecological stability within nested network patterns (Montoya et al., 2006).

Second, from a belowground perspective, the conception of anthrosols within *beige* corridors may be rethought to reach suitable soil properties (i.e., biotic and abiotic conditions, including chemical and physical properties) for the establishment and the expansion of physical ECM links among plants. Accommodations may encompass a reduction of soil sealing area, the use of permeable sealing materials, the edification of stratified and organic-enriched pedological profiles, and controlled ECM inoculation by native fungi (Figure 3). Ultimately, combining above- and belowground facets of *beige* corridors may be in line with the rewilding projects that gain popularity in landscape planning.

## Author Contributions

FR, LA, and CV wrote the manuscript and designed the figures. All authors contributed to the article and approved the submitted version.

## Funding

This research was funded by the French Agency for Research and Technology (ANRT; contract CIFRE n°2020/0269).

## Conflict of Interest

The authors declare that the research was conducted in the absence of any commercial or financial relationships that could be construed as a potential conflict of interest.

## Publisher’s Note

All claims expressed in this article are solely those of the authors and do not necessarily represent those of their affiliated organizations, or those of the publisher, the editors and the reviewers. Any product that may be evaluated in this article, or claim that may be made by its manufacturer, is not guaranteed or endorsed by the publisher.

## Figures and Tables

**Table 1:** 

Alpha-diversity	Diversity of co-occurring organisms at local scale. Species richness is a widely used measurement of alpha diversity as a taxonomical facet of biodiversity. Contrastingly, the beta-diversity of metacommunities characterizes the level of composition differences between communities.
Anthropogenic	Refers to processes, assemblies and ecosystem compartments driven by, or resulting from human activity. Anthropogenic landscapes typically consist of vegetation mosaics induced by long-term human activity (forestry, pastoralism, fire regime, etc.). Anthropogenic soils include pedological profiles that are physico-chemically altered by long-term anthropic pressures as well as matrices of artificial materials from urban/industrial origin (Anthrosols).
Cultivated forest	Consists of highly diversified physiognomies of vegetation dominated by trees, originated from either natural process (spontaneous establishment of seedlings) of plantations, where human practices drive the structure, the composition and the dynamics of tree populations. Cultivated forests encompass forestry-based managed forests and agroforests, including planted orchards and multifunctional tree savannas.
Endophytic lifeforms	Micro-organisms, including bacteria and filamentous fungi, leaving inside plant tissues (roots, seeds, leaves, bark, etc.) where they accomplish a part or their entire biological cycle. Recent research shows the ability of ectomycorrhizal fungi (e.g., Tuber melanosporum) to colonize root tissues of AM host as endophytes.
Network topology	Set of properties characterizing an interaction network, used to infer ecological/evolutionary hypotheses, and based on the analysis of the distribution of links between the objects (nodes) constituting the network.
Sealed soils	Soils that have sequestered in the mid-term by a cover of impermeable materials (e.g., tar) hindering pedogenesis.
